# Supplementary material for: Patterns of Intron Gain and Loss in Fungi
Source: PLoS Biol. 2004 Nov 30;2(12):e422. doi: 10.1371/journal.pbio.0020422 (PMC532390; doi:10.1371/journal.pbio.0020422)
Supplement: Table S1 — Also available at http://genes.mit.edu/NielsenEtAl/. (4.3 MB ZIP). [file pbio.0020422.st001.zip › NielsenEtAl/html/211.html]

AN4806.1.NCU04061.1.MG01293.1.FG11045.1


```
 CLUSTAL W (1.82) Multiple Sequence Alignments - Introns Inserted


Sequence 1: MG01293.1	191 aa
Sequence 2: FG11045.1	178 aa
Sequence 3: AN4806.1	188 aa
Sequence 4: NCU04061.1	185 aa
Alignment Length: 203 aa
Number Identitical Residues: 78 aa
Alignment Score (without introns) 4241


MG01293.1 	MACRYYPATASYPSPLKGFENLPPLSE~EKDGDGKSLRNEQTGVRSKAYEEFVDPLDRGK
NCU04061.1	MP----SQRPTYKSPLEGYENAPPLPT~EKAEDGKSLLNNQTGVLSSAYEKFPEPLDNGR
FG11045.1 	MG---DASVFKYPSPLTGYEDAPPLPN~DMAEDGKSYVN-PAAKKSEAYEKFIDPFDQTP
AN4806.1  	MT---DQFAYTYPSPLVGYENLEPLPE2ERNDDGKSLKNPQHGILSKAYEEFPDPLSKGR
          	*         .* *** *:*:  **.  :   ****  *   .  *.***:* :*:..  

MG01293.1 	RGGF2DVHIYYFH~ASEDQTKYAKELYERIRREF1PELRIYKFWDRPVGPHPVAMFEVNL
NCU04061.1	RGAF2DVHIYHLP~NHPAQARYAAELHSRIRHEF~PELRIYTFWSQPVGPHPIGMFEVNI
FG11045.1 	RGAF2DVHIYYFQ~GNKEQTKYAKELWERIRREF1PELRTYKVWDGPIGPHPVAMFEVNI
AN4806.1  	RGGF~DIHIYHFQ0NNPDQVAYARALWERIRREF1PELRIYTFWDKPVGPHPVAMFEVNL
          	**.* *:***::      *. **  * .***:** **** *..*. *:****:.*****:

MG01293.1 	FTPAQFGAFVAWLAVWRGPLSALVHPN----TIAGPGED-QIEAERRDHSQRAIWMGERL
NCU04061.1	FTPAQLGAFVGWLAVWRGPLSVLIHPNTTEKTISEGGEISEAEKEARNHTDKAIWMGEKV
FG11045.1 	FTPAQFGAFIPWLNVWRGPLSALVHPN----IIPEEGVD-RWASMKRDHLERAIWMGERI
AN4806.1  	FTPAQFGAFIPWLVIYRGPLSVLVHPN----TEAHEDE--------RNHTQRAMWLGDRI
          	*****:***: ** ::*****.*:***      .  .         *:* ::*:*:*:::

MG01293.1 	PLDLDLFAKMAERKASAGNS-------
NCU04061.1	VLNTDMFWEVMDR--------------
FG11045.1 	PLDLSLFNNQS----------------
AN4806.1  	PLDLRVFKIMKEKERVEREKNENKEKL
          	 *:  :*    . .    ........
```
